# Heat waves: a hot topic in climate change research

**DOI:** 10.1007/s00704-021-03758-y

**Published:** 2021-09-03

**Authors:** Werner Marx, Robin Haunschild, Lutz Bornmann

**Affiliations:** 1 Max Planck Institute for Solid State Research, Heisenbergstr. 1, 70569 Stuttgart, Germany; 2 Science Policy and Strategy Department, Max Planck Society, Administrative Headquarters, Hofgartenstr. 8, 80539 Munich, Germany

## Abstract

Research on heat waves (periods of excessively hot weather, which may be accompanied by high humidity) is a newly emerging research topic within the field of climate change research with high relevance for the whole of society. In this study, we analyzed the rapidly growing scientific literature dealing with heat waves. No summarizing overview has been published on this literature hitherto. We developed a suitable search query to retrieve the relevant literature covered by the Web of Science (WoS) as complete as possible and to exclude irrelevant literature (*n* = 8,011 papers). The time evolution of the publications shows that research dealing with heat waves is a highly dynamic research topic, doubling within about 5 years. An analysis of the thematic content reveals the most severe heat wave events within the recent decades (1995 and 2003), the cities and countries/regions affected (USA, Europe, and Australia), and the ecological and medical impacts (drought, urban heat islands, excess hospital admissions, and mortality). An alarming finding is that the limit for survivability may be reached at the end of the twenty-first century in many regions of the world due to the fatal combination of rising temperatures and humidity levels measured as “wet-bulb temperature” (WBT). Risk estimation and future strategies for adaptation to hot weather are major political issues. We identified 104 citation classics, which include fundamental early works of research on heat waves and more recent works (which are characterized by a relatively strong connection to climate change).

## Introduction

As a consequence of the well-documented phenomenon of global warming, climate change has become a major research field in the natural and medical sciences, and more recently also in the social and political sciences. The scientific community has contributed extensively to a comprehensive understanding of the earth’s climate system, providing various data and projections on the future climate as well as on the effects and risks of anticipated global warming (IPCC 2014; CSSR 2017; NCA4 2018; and the multitude of references cited therein). During recent decades, climate change has also become a major political, economic, and environmental issue and a central theme in political and public debates.

One consequence of global warming is the increase of extreme weather events such as heat waves, droughts, floods, cyclones, and wildfires. Some severe heat waves occurring within the last few decades made heat waves a hot topic in climate change research, with “hot” having a dual meaning: high temperature and high scientific activity. “More intense, more frequent, and longer lasting heat waves in the twenty-first century” is the title of a highly cited paper published 2004 in *Science* (Meehl and Tebaldi 2004). This title summarizes in short what most climate researchers anticipate for the future. But what are heat waves (formerly also referred to as “heatwaves”)? In general, a heat wave is a period of excessively hot weather, which may be accompanied by high humidity. Since heat waves vary according to region, there is no universal definition, but only definitions relative to the usual weather in the area and relative to normal temperatures for the season. The World Meteorological Organization (WMO) defines a heat wave as 5 or more consecutive days of prolonged heat in which the daily maximum temperature is higher than the average maximum temperature by 5 °C (9 °F) or more (https://www.britannica.com/science/heat-wave-meteorology).

Europe, for example, has suffered from a series of intense heat waves since the beginning of the twenty-first century. According to the World Health Organization (WHO) and various national reports, the extreme 2003 heat wave caused about 70,000 excess deaths, primarily in France and Italy. The 2010 heat wave in Russia caused extensive crop loss, numerous wildfires, and about 55,000 excess deaths (many in the city of Moscow). Heat waves typically occur when high pressure systems become stationary and the winds on their rear side continuously pump hot and humid air northeastward, resulting in extreme weather conditions. The more intense and more frequently occurring heat waves cannot be explained solely by natural climate variations and without human-made climate change (IPCC 2014; CSSR 2017; NCA4 2018). Scientists discuss a weakening of the polar jet stream caused by global warming as a possible reason for an increasing probability for the occurrence of stationary weather, resulting in heavy rain falls or heat waves (Broennimann et al. 2009; Coumou et al. 2015; Mann 2019). This jet stream is one of the most important factors for the weather in the middle latitude regions of North America, Europe, and Asia.

Until the end of the twentieth century, heat waves were predominantly seen as a recurrent meteorological fact with major attention to drought, being almost independent from human activities and unpredictable like earthquakes. However, since about 1950, distinct changes in extreme climate and weather events have been increasingly observed. Meanwhile, climate change research has revealed that these changes are clearly linked to the human influence on the content of greenhouse gases in the earth’s atmosphere. Climate-related extremes, such as heat waves, droughts, floods, cyclones, and wildfires, reveal significant vulnerability to climate change as a result of global warming.

In recent years, research on heat waves has been established as an emerging research topic within the large field of current climate change research. Bibliometric analyses are very suitable in order to have a systematic and quantitative overview of the literature that can be assigned to an emerging topic such as research dealing with heat waves (e.g., Haunschild et al. 2016). No summarizing overview on the entire body of heat wave literature has been published until now. However, a bibliometric analysis of research on urban heat islands as a more specific topic in connection with heat waves has been performed (Huang and Lu 2018).

In this study, we analyzed the publications dealing with heat waves using appropriate bibliometric methods and tools. First, we determined the amount and time evolution of the scientific literature dealing with heat waves. The countries contributing the most papers are presented. Second, we analyzed the thematic content of the publications via keywords assigned by the WoS. Third, we identified the most important (influential) publications (and also the historical roots). We identified 104 citation classics, which include fundamental early works and more recent works with a stronger connection to climate change.

## Heat waves as a research topic

The status of the current knowledge on climate change is summarized in the *Synthesis Report of the Fifth Assessment Report (AR5)* of the *Intergovernmental Panel on Climate Change (IPCC)* (IPCC 2014, https://www.ipcc.ch/report/ar5/syr/). This panel is the United Nations body for assessing the science related to climate change. The *Synthesis Report* is based on the reports of the three *IPCC Working Groups*, including relevant *Special Reports*. In its *Summary for Policymakers*, it provides an integrated view of climate change as the final part of the *Fifth Assessment Report* (IPCC 2014, https://www.ipcc.ch/site/assets/uploads/2018/02/AR5_SYR_FINAL_SPM.pdf).

In the chapter *Extreme Events*, it is stated that “changes in many extreme weather and climate events have been observed since about 1950. Some of these changes have been linked to human influences, including a decrease in cold temperature extremes, an increase in warm temperature extremes, an increase in extreme high sea levels and an increase in the number of heavy precipitation events in a number of regions … It is very likely that the number of cold days and nights has decreased and the number of warm days and nights has increased on the global scale. It is likely that the frequency of heat waves has increased in large parts of Europe, Asia and Australia. It is very likely that human influence has contributed to the observed global scale changes in the frequency and intensity of daily temperature extremes since the mid-twentieth century. It is likely that human influence has more than doubled the probability of occurrence of heat waves in some locations” (p. 7–8). Under *Projected Changes*, the document summarizes as follows: “Surface temperature is projected to rise over the twenty-first century under all assessed emission scenarios. It is very likely that heat waves will occur more often and last longer, and that extreme precipitation events will become more intense and frequent in many regions” (p. 10).

With regard to the USA, the *Climate Science Special Report* of the *U.S. Global Change Research Program* (CSSR 2017, https://science2017.globalchange.gov/) mentions quite similar observations and states unambiguously in its *Fourth National Climate Assessment (Volume I)* report (https://science2017.globalchange.gov/downloads/CSSR2017_FullReport.pdf) under *Observed Changes in Extremes* that “the frequency of cold waves has decreased since the early 1900s, and the frequency of heat waves has increased since the mid-1960s (very high confidence). The frequency and intensity of extreme heat and heavy precipitation events are increasing in most continental regions of the world (very high confidence). These trends are consistent with expected physical responses to a warming climate [p. 19]. Heavy precipitation events in most parts of the United States have increased in both intensity and frequency since 1901 (high confidence) [p. 20]. There are important regional differences in trends, with the largest increases occurring in the northeastern United States (high confidence). Recent droughts and associated heat waves have reached record intensity in some regions of the United States … (very high confidence) [p. 21]. Confidence in attribution findings of anthropogenic influence is greatest for extreme events that are related to an aspect of temperature” (p. 123).

Among the key findings in the chapter on temperature changes in the USA, the report states that “there have been marked changes in temperature extremes across the contiguous United States. The frequency of cold waves has decreased since the early 1900s, and the frequency of heat waves has increased since the mid-1960s (very high confidence). Extreme temperatures in the contiguous United States are projected to increase even more than average temperatures. The temperatures of extremely cold days and extremely warm days are both expected to increase. Cold waves are projected to become less intense while heat waves will become more intense (very high confidence) [p. 185]. Most of this methodology as applied to extreme weather and climate event attribution, has evolved since the European heat wave study of Stott et al.” (p. 128).

Heat waves are also discussed in the *Fourth National Climate Assessment (Volume II)* report (NCA4 2018, https://nca2018.globalchange.gov/). The *Report-in-Brief* (https://nca2018.globalchange.gov/downloads/NCA4_Report-in-Brief.pdf) for example states: “More frequent and severe heat waves and other extreme events in many parts of the United States are expected [p. 38]. Heat waves and heavy rainfalls are expected to increase in frequency and intensity [p. 93]. The season length of heat waves in many U.S. cities has increased by over 40 days since the 1960s [p. 30]. Cities across the Southeast are experiencing more and longer summer heat waves [p. 123]. Exposure to hotter temperatures and heat waves already leads to heat-associated deaths in Arizona and California. Mortality risk during a heat wave is amplified on days with high levels of ground-level ozone or particulate air pollution” (p. 150).

In summary, climate change research expects more frequent and more severe heat wave events as a consequence of global warming. It is likely that the more frequent and longer lasting heat waves will significantly increase excess mortality, particularly in urban regions with high air pollution. Therefore, research around heat waves will become increasingly important and is much more than a temporary research fashion.

## Methodology

### Dataset used

This analysis is based on the relevant literature retrieved from the following databases accessible under the Web of Science (WoS) of Clarivate Analytics: Web of Science Core Collection: Citation Indexes, Science Citation Index Expanded (SCI-EXPANDED), Social Sciences Citation Index (SSCI), Arts & Humanities Citation Index (A&HCI), Conference Proceedings Citation Index—Science (CPCI-S), Conference Proceedings Citation Index—Social Science & Humanities (CPCI-SSH), Book Citation Index—Science (BKCI-S), Book Citation Index—Social Sciences & Humanities (BKCI-SSH), Emerging Sources Citation Index (ESCI).

We applied the search query given in Appendix 1 to cover the relevant literature as completely as possible and to exclude irrelevant literature. We practiced an iterative query optimization by identifying and excluding the WoS subject categories with most of the non-relevant papers. For example, heat waves are also mentioned in the field of materials science but have nothing to do with climate and weather phenomena. Unfortunately, WoS obviously assigned some heat wave papers related to climate to materials science-related subject categories. Therefore, these subject categories were not excluded. By excluding the other non-relevant subject categories, 597 out of 8,568 papers have been removed, resulting in a preliminary publication set of 7,971 papers (#2 of the search query). But this is no safe method, since the excluded categories may well include some relevant papers. Therefore, we have combined these 597 papers with search terms related to climate or weather and retrieved 62 relevant papers in addition, which we added to our preliminary paper subset, eventually receiving 8,033 publications (#3 to #5 of the search query).

Commonly, publication sets for bibliometric analyses are limited to articles, reviews, and conference proceedings as the most relevant document types and are restricted to complete publication years. In this study, however, we have included all relevant WoS document types for a better literature coverage of the research topic analyzed. For example, conference meetings and early access papers may well be interesting for the content analysis of the literature under study. Such literature often anticipates important results, which are published later as regular articles. Furthermore, we have included the literature until the date of search for considering the recent rapid growth of the field. Our search retrieved a final publication set of 8,011 papers indexed in WoS until the date of search (July 1, 2021) and dealing with heat waves (#6 of the search query). We have combined this publication set with climate change-related search terms from a well-proven search query (Haunschild et al. 2016) resulting in 4,588 papers dealing with heat waves in connection with climate change or global warming (# 11 of the search query). Also, we have selected a subset of 2,373 papers dealing with heat waves and mortality (#13 of the search query). The complete WoS search query is given in Appendix 1.

The final publication set of 8,011 papers dealing with heat waves still contains some non-relevant papers primarily published during the first half of the twentieth century, such as some *Nature* papers within the WoS category *Multidisciplinary Sciences*. Since these papers are assigned only to this broad subject category and have no abstracts and no keywords included, they cannot be excluded using the WoS search and refinement functions. We do not expect any bias through these papers, because their keywords do not appear in our maps. Also, they normally contain very few (if any) cited references, which could bias/impact our reference analysis.

### Networks

We used the VOSviewer software (Van Eck and Waltman 2010) to map co-authorship with regard to the countries of authors (88 countries considered) of the papers dealing with heat waves (www.vosviewer.com). The map of the cooperating countries presented is based on the number of joint publications. The distance between two nodes is proportionate to the number of co-authored papers. Hence, largely cooperating countries are positioned closer to each other. The size of the nodes is proportionate to the number of papers published by authors of the specific countries.

The method that we used for revealing the thematic content of the publication set retrieved from the WoS is based on the analysis of keywords. For better standardization, we chose the keywords allocated by the database producer (keywords plus) rather than the author keywords. We also used the VOSviewer for mapping the thematic content of the 104 key papers selected by reference analysis. This map is also based on keywords plus.

The term maps (keywords plus) are based on co-occurrence for positioning the nodes on the maps. The distance between two nodes is proportionate to the co-occurrence of the terms. The size of the nodes is proportionate to the number of papers with a specific keyword. The nodes on the map are assigned by VOSviewer to clusters based on a specific cluster algorithm (the clusters are highlighted in different colors). These clusters identify closely related (frequently co-occurring) nodes, where each node is assigned to only one cluster.

### Reference Publication Year Spectroscopy

A bibliometric method called “Reference Publication Year Spectroscopy” (RPYS, Marx et al. 2014) in combination with the tool CRExplorer (http://www.crexplorer.net, Thor et al. 2016a, b) has proven useful for exploring the cited references within a specific publication set, in order to detect the most important publications of the relevant research field (and also the historical roots). In recent years, several studies have been published, in which the RPYS method was basically described and applied (Marx et al. 2014; Marx and Bornmann 2016; Comins and Hussey 2015). In previous studies, Marx et al. have analyzed the roots of research on global warming (Marx et al. 2017a), the emergence of climate change research in combination with viticulture (Marx et al. 2017b), and tea production (Marx et al. 2017c) from a quantitative (bibliometric) perspective. In this study, we determined which references have been most frequently cited by the papers dealing with heat waves.

RPYS is based on the assumption that peers produce a useful database by their publications, in particular by the references cited therein. This database can be analyzed statistically with regard to the works most important for their specific research field. Whereas individual scientists judge their research field more or less subjectively, the overall community can deliver a more objective picture (based on the principle of “the wisdom of the crowds”). The peers effectively “vote” via their cited references on which works turned out to be most important for their research field (Bornmann and Marx 2013). RPYS implies a normalization of citation counts (here: reference counts) with regard to the research area and the time of publication, which both impact the probability to be cited frequently. Basically, the citing and cited papers analyzed were published in the same research field and the reference counts are compared with each other only within the same publication year.

RPYS relies on the following observation: the analysis of the publication years of the references cited by all the papers in a specific research topic shows that publication years are not equally represented. Some years occur particularly frequently among the cited references. Such years appear as distinct peaks in the distribution of the reference publication years (i.e., the RPYS spectrogram). The pronounced peaks are frequently based on a few references that are more frequently cited than other references published in the same year. The frequently cited references are—as a rule—of specific significance to the research topic in question (here: heat waves) and the earlier references among them represent its origins and intellectual roots (Marx et al. 2014).

The RPYS changes the perspective of citation analysis from a times cited to a cited reference analysis (Marx and Bornmann 2016). RPYS does not identify the most highly cited papers of the publication set being studied (as is usually done by bibliometric analyses in research evaluation). RPYS aims to mirror the knowledge base of research (here: on heat waves).

With time, the body of scientific literature of many research fields is growing rapidly, particularly in climate change research (Haunschild et al. 2016). The growth rate of highly dynamic research topics such as research related to heat waves is even larger. As a consequence, the number of potentially citable papers is growing substantially. Toward the present, the peaks of individual publications lie over a broad continuum of newer publications and are less numerous and less pronounced. Due to the many publications cited in the more recent years, the proportion of individual highly cited publications in specific reference publication years falls steadily. Therefore, the distinct peaks in an RPYS spectrogram reveal only the most highly cited papers, in particular the earlier references comprising the historical roots. Further inspection and establishing a more entire and representative list of highly cited works requires consulting the reference table provided by the CRExplorer. The most important references within a specific reference publication year can be identified by sorting the cited references according to the reference publication year (RPY) and subsequently according to the number of cited references (N_CR) in a particular publication year.

The selection of important references in RPYS requires the consideration of two opposing trends: (1) the strongly growing number of references per reference publication year and (2) the fall off near present due to the fact that the newest papers had not sufficient time to accumulate higher citation counts. Therefore, we decided to set different limits for the minimum number of cited references for different periods of reference publication years (1950–1999: N_CR ≥ 50, 2000–2014: N_CR ≥ 150, 2015–2020: N_CR ≥ 100). This is somewhat arbitrary, but is helpful in order to adapt and limit the number of cited references to be presented and discussed.

In order to apply RPYS, all cited references (*n* = 408,247) of 216,932 unique reference variants have been imported from the papers of our publication set on heat waves (*n* = 8,011). The cited reference publication years range from 1473 to 2021. We removed all references (297 different cited reference variants) with reference publication years prior to 1900. Due to the very low output of heat wave-related papers published before 1990, no relevant literature published already in the nineteenth century can be expected. Also, global warming was no issue before 1900 since the *Little Ice Age* (a medieval cold period) lasted until the nineteenth century. The references were sorted according to RPY and N_CR for further inspection.

The CRExplorer offers the possibility to cluster and merge variants of the same cited reference (Thor et al. 2016a, b). We clustered and merged the associated reference variants in our dataset (which are mainly caused by misspelled references) using the corresponding CRExplorer module, clustering the reference variants via volume and page numbers and subsequently merging aggregated 374 cited references (for more information on using the CRExplorer see “guide and datasets” at www.crexplorer.net).

After clustering and merging, we applied a further cutback: to focus the RPYS on the most pronounced peaks, we removed all references (*n* = 212,324) with reference counts below 10 (resulting in a final number of 3,937 cited references) for the detection of the most frequently cited works. A minimum reference count of 10 has proved to be reasonable, in particular for early references (Marx et al. 2014). The cited reference publication years now range from 1932 to 2020.

## Results

In this study, we have considered all relevant WoS document types for a preferably comprehensive coverage of the literature of the research topic analyzed. The vast majority of the papers of our publication set, however, have been assigned to the document types “article” (*n* = 6.738, 84.1%), “proceedings paper” (*n* = 485, 6.1%), and “review” (*n* = 395 papers, 4.9%). Note that some papers belong to more than one document type.

### Time evolution of literature

**Fig. 1:**
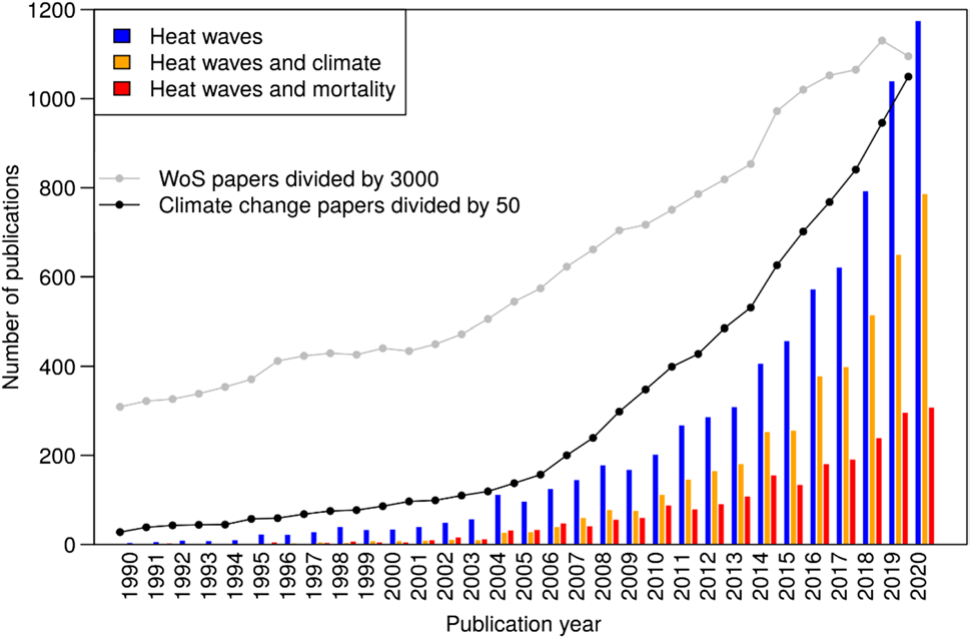
Time evolution of the overall number of heat wave publications, of heat wave publications in connection with climate change, and of heat wave publications in connection with mortality, each between 1990 and 2020. For comparison, the overall number of publications (scaled down) in the field of climate change research and the total number of publications covered by the WoS database (scaled down, too) are included

In Fig. 1, the time evolution between 1990 and 2020 of the publications dealing with heat waves is shown (there are only 109 pre-1990 publications dealing with heat waves and covered by the WoS).

According to Fig. 1, research dealing with heat waves is a highly dynamic research topic, currently doubling within about 5 years. The number of papers published per year shows a strong increase: since around 2000, the publication output increased by a factor of more than thirty, whereas in the same period, the overall number of papers covered by the WoS increased only by a factor of around three. Also, the portion of heat wave papers dealing with climate change increased substantially: from 16.1 in the period 1990–1999 to 25.7% in 2000, reaching 66.9% in 2020. The distinct decrease of the overall number of papers covered by the WoS between 2019 and 2020 might be a result of the Covid-19 pandemic.

With regard to the various impacts of heat waves, excess mortality is one of the most frequently analyzed and discussed issues in the scientific literature (see below). Whereas the subject specific literature on heat waves increased from 2000 to 2020 by a factor of 33.6, literature on heat waves dealing with mortality increased from 2000 to 2020 by a factor of 51.5. The dynamics of the research topic dealing with heat waves is mirrored by the WoS *Citation Report*, which shows the time evolution of the overall citation impact of the papers of the publication set (not presented). The citation report curve shows no notable citation impact before 2005, corresponding to the increase of the publication rate since about 2003 as shown in Fig. 1.

### Countries of authors

In Table 1, the number of papers assigned to the countries of authors with more than 100 publications dealing with heat waves is presented, showing the national part of research activities on this research topic. For comparative purposes, the percentage of overall papers in WoS of each country is shown. As a comparison with the overall WoS, we only considered WoS papers published between 2000 and 2020, because the heat wave literature started to grow substantially around 2000.

**Table 1:** Top countries of authors with more than 100 papers dealing with heat waves up to the date of the search

Country of authors	#Papers	%Papers heat waves	%Papers overall in WoS
USA	2081	26.0	27.4
Australia	1026	12.8	3.1
Peoples Republic of China	965	12.0	12.1
England	760	9.5	6.7
Germany	737	9.2	6.3
France	638	8.0	4.3
Italy	536	6.7	3.9
Spain	506	6.3	3.1
Switzerland	361	4.5	1.6
Canada	356	4.4	4.0
India	236	2.9	3.3
Netherlands	227	2.8	2.2
South Korea	206	2.6	2.5
Sweden	206	2.6	1.4
Portugal	204	2.5	0.7
Belgium	176	2.2	1.2
Japan	168	2.1	5.2
Greece	163	2.0	0.7
Russia	149	1.9	2.1
Poland	141	1.8	1.4
Austria	137	1.7	0.9
Czech Republic	130	1.6	0.7
Denmark	119	1.5	0.9
South Africa	119	1.5	0.6
Brazil	116	1.4	2.1
Scotland	106	1.3	1.0

**Fig. 2:**
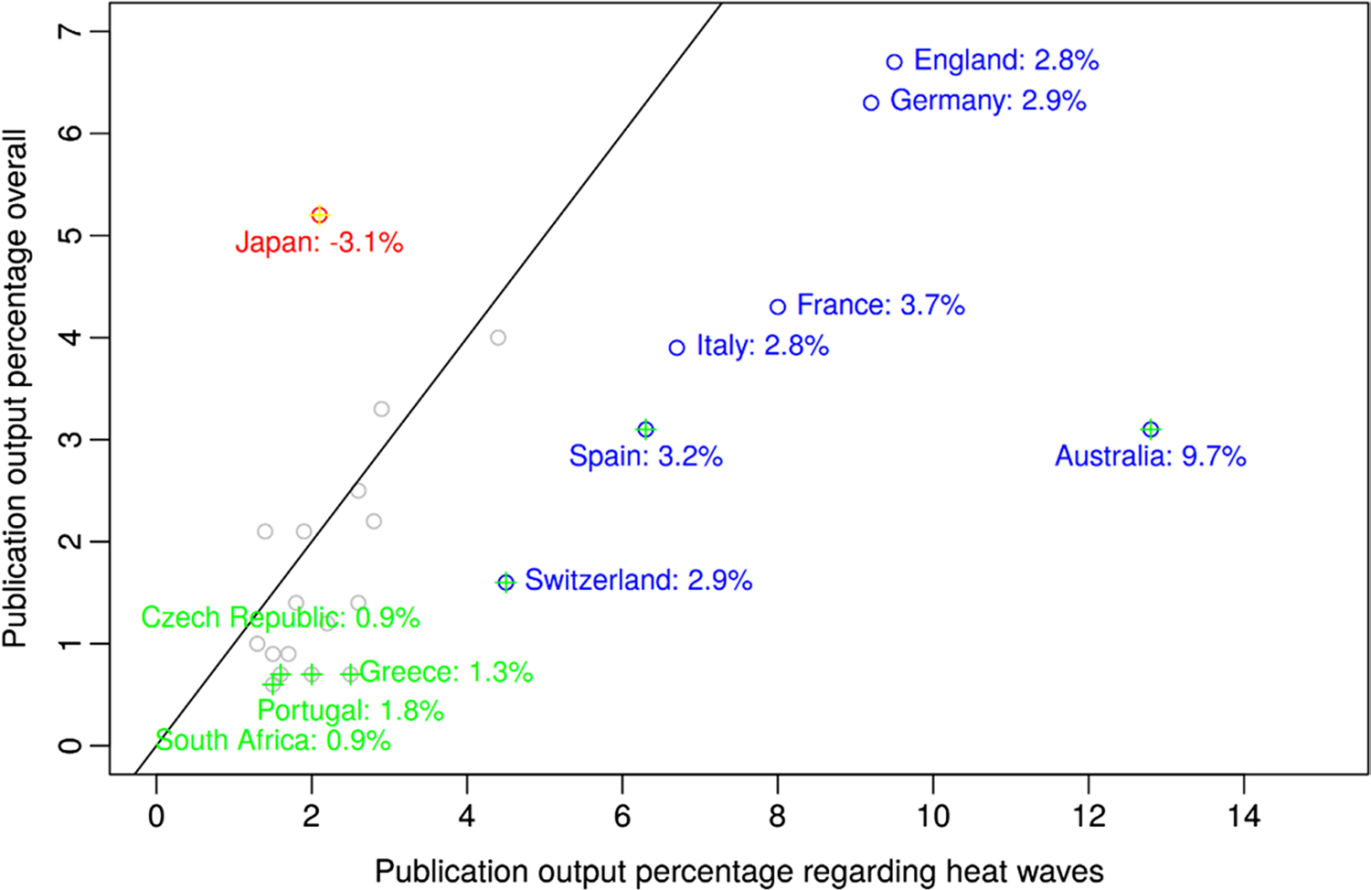
Publication percentages of countries in Table 1. Countries with large deviations between heat wave output and overall WoS output are labeled. Countries with an absolute percentage of more than two percentage points higher (lower) in heat wave research than in overall WoS output are marked blue (red). Countries with a relative percentage at least twice as high (at most half as much) in heat wave research than in overall WoS output are marked green (yellow)

The country-specific percentages from Table 1 are visualized in Fig. 2. Selected countries are labeled. Countries with a higher relative percentage of more than two percentage points in heat wave research than in WoS overall output are marked blue (blue circle). Countries with a relative percentage at least twice as high in heat wave research than in overall WoS output are marked green (green cross), whereas countries with a relative percentage at most half as much in heat wave research than in overall WoS output are marked with a yellow cross. Only Japan has a much lower output in heat wave research than in WoS overall output, as indicated by the red circle and yellow cross. Most countries are clustered around the bisecting line and are marked gray (gray circle). China and the USA are outside of the plot region. Both countries are rather close to the bisecting line. Some European countries show a much larger activity in heat wave research than in overall WoS output. Australia shows the largest difference and ratio in output percentages as shown by the blue circle and green cross.

The results mainly follow the expectations of such bibliometric analyses, with one distinct exception: Australia increasingly suffers from extreme heat waves and is comparatively active in heat wave research—compared with its proportion of scientific papers in general. The growth factor of the Australian publication output since 2010 is 8.5, compared to 5.3 for the USA and 3.3 for Germany.

**Fig. 3:**
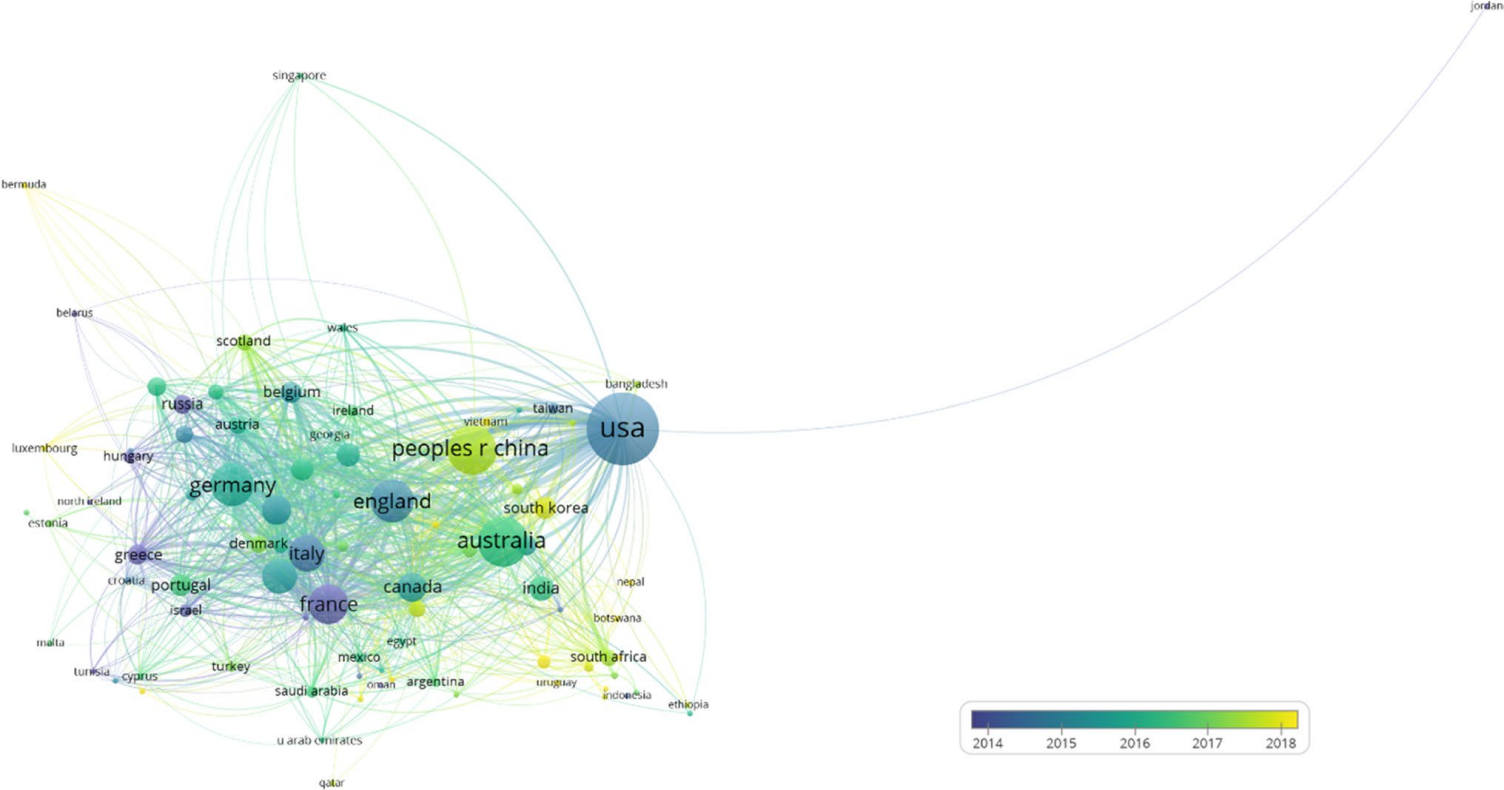
Co-authorship overlay map with regard to the countries of authors and their average publication years from the 8,011 papers dealing with heat waves. The minimum number of co-authored publications of a country is 5; papers with more than 25 contributing countries are neglected; of the 135 countries, 89 meet the threshold, and 88 out of 89 countries are connected and are considered (one country, Armenia, that is disconnected from the network has been removed). The co-authorship network of a single country can be depicted by clicking on the corresponding node in the interactive map. Readers interested in an in-depth analysis can use VOSviewer interactively and zoom into the map via the following URL: https://tinyurl.com/3ywkwv8t

Figure 3 shows the co-authorship network with regard to the countries of authors of the papers dealing with heat waves using the VOSviewer software.

According to Fig. 3 and in accordance with Table 1, the USA is most productive in heat wave research. This is not unexpected, because the US publication output is at the top for most research fields. However, this aside, the USA has been heavily affected by heat wave events and is leading with regard to the emergence of the topic. Australia appears as another major player and is strongly connected with the US publications within the co-authorship network and thus appears as a large node near the US node in the map. Next, the leading European countries England, France, Germany, Italy, and Spain appear.

The overlay version of the map includes the time evolution of the research activity in the form of coloring of the nodes. The map shows the mean publication year of the publications for each specific author country. As a consequence, the time span of the mean publication years ranges only from 2014 to 2018. Nevertheless, the early activity in France and the USA and the comparatively recent activity in Australia and China, with the European countries in between, become clearly visible.

### Topics of the heat wave literature

**Fig. 4:**
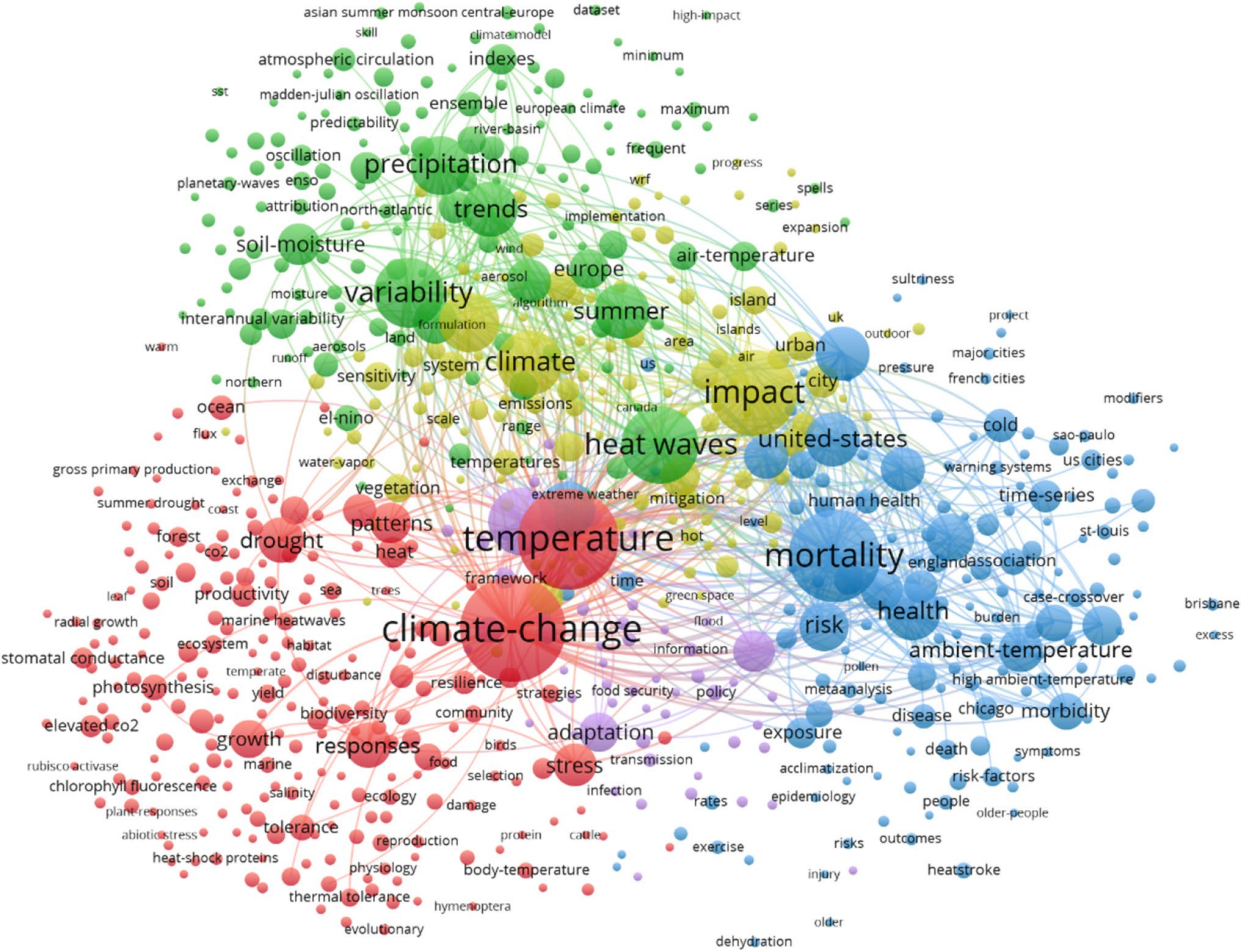
Co-occurrence network map of the keywords plus from the 8,011 papers dealing with heat waves for a rough analysis of the thematic content. The minimum number of occurrences of keywords is 10; of the 10,964 keywords, 718 meet the threshold. Readers interested in an in-depth analysis can use VOSviewer interactively and zoom into the map via the following URL: https://tinyurl.com/enrdbw

Figure 4 shows the keywords (keywords plus) map for revealing the thematic content of our publication set using the VOSviewer software. This analysis is based on the complete publication set (*n* = 8,011). The minimum number of occurrences of keywords is 10; of the 10,964 keywords, 718 keywords met the threshold. For each of the 718 keywords, the total strength of the co-occurrence links with other keywords was calculated. The keywords with the greatest total link strength were selected for presentation in the map.

According to Fig. 4, the major keywords are the following: climate change, temperature, mortality, impact, heat waves (searched), and variability. The colored clusters identify closely related (frequently co-occurring) nodes. The keywords marked red roughly originate from fundamental climate change research focused on the hydrological cycle (particularly on drought), the keywords of the green cluster are around heat waves and moisture or precipitation, the keywords marked blue result from research concerning impacts of heat waves on health, the keywords marked yellow are focused on the various other impacts of heat waves, and the keywords of the magenta cluster are around adaptation and vulnerability in connection with heat waves.

The clustering by the VOSviewer algorithm provides basic categorizations, but many related keywords also appear in different clusters. For example, severe heat wave events are marked in different colors. For a better overview of the thematic content of the publications dealing with heat waves, we have assigned the keywords of Fig. 4 (with a minimum number of occurrences of 50) to ten subject categories (each arranged in the order of occurrence):

1. Countries/regions: United-States, Europe, France, China, Pacific, Australia, London, England
2. Cities: cities, city, US cities, Chicago, communities
3. Events: 2003 heat-wave, 1995 heat-wave
4. Impacts: impact, impacts, air-pollution, drought, soil-moisture, exposure, heat-island, urban, islands, photosynthesis, pollution, heat-island, air-quality, environment, precipitation extremes, biodiversity, emissions
5. Politics: risk, responses, vulnerability, adaptation, management, mitigation, risk-factors, scenarios
6. Biology: vegetation, forest, diversity, stomatal conductance
7. Medicine: mortality, health, stress, deaths, morbidity, hospital admissions, public-health, thermal comfort, population, heat, sensitivity, human health, disease, excess mortality, heat-stress, heat-related mortality, comfort, behavior, death, stroke
8. Climate research: climate change, temperature, climate, model, simulation, energy, projections, simulations, cmip5, ozone, el-nino, parametrization, elevated CO_2_, models, climate variability, carbon, carbon-dioxide
9. Meteorology: heat waves, variability, precipitation, summer, heat-wave, weather, ambient-temperature, waves, extremes, wave, cold, water, rainfall, circulation, heat, air-temperature, extreme heat, climate extremes, heatwaves, temperature extremes, temperatures, temperature variability, high-temperature, ocean, extreme temperatures, atmospheric circulation, interannual variability, sea-surface temperature, oscillation, surface temperature, surface
10. Broader terms (multi-meaning): trends, events, patterns, growth, performance, time-series, indexes, system, dynamics, association, index, tolerance, productivity, ensemble, resilience, increase, quality, prediction, frequency, particulate matter, future, framework, 20^th^-century, time, reanalysis, systems

Although allocated by the database provider, the keywords are not coherent. For example, the same keyword may appear as singular or plural, and complex keywords are written with and without hyphens.

**Fig. 5:**
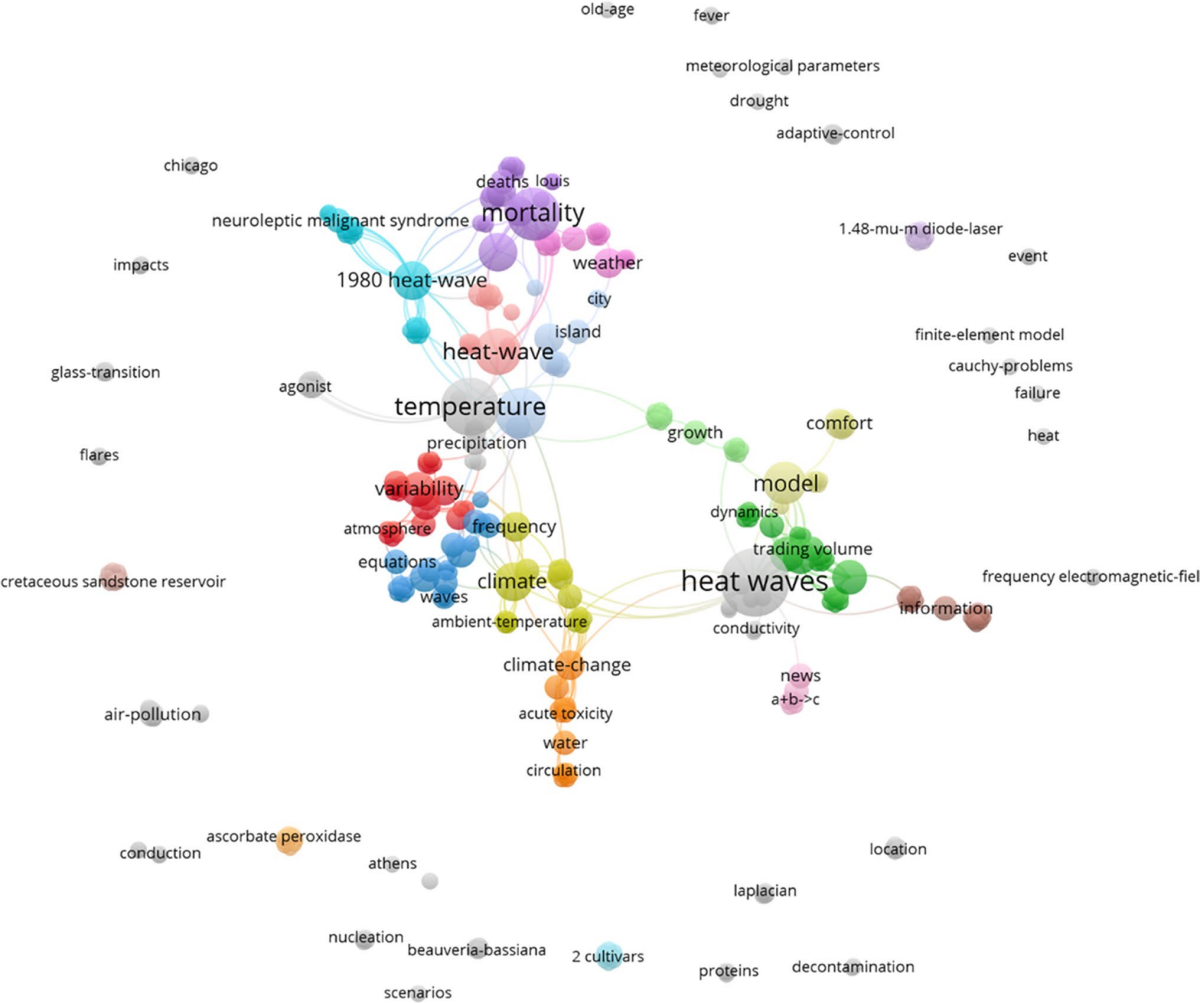
Co-occurrence network map of the keywords plus from the 297 pre-2000 papers dealing with heat waves for a rough analysis of the thematic content. The minimum number of occurrences of keywords is 1; of the 389 keywords, 277 keywords are connected, and all items are shown. Readers interested in an in-depth analysis can use VOSviewer interactively and zoom into the map via the following URL: https://tinyurl.com/u2zzr399

In order to compare the thematic content of the complete publication set with the earlier literature on heat waves, we have analyzed the pre-2000 publications (*n* = 297) separately. Figure 5 shows the keywords (keywords plus) map for revealing the thematic content of the pre-2000 papers.

The major nodes in Fig. 5 are heat waves (searched), temperature, United States, and mortality, with climate change appearing only as a smaller node here. Obviously, the connection between heat waves and climate change was not yet pronounced, which can also be seen from Fig. 1. Compared with Fig. 4, the thematic content of the clusters is less clear and the clusters presented in Fig. 5 can hardly be assigned to specific research areas. For a better overview of the thematic content of the early publications dealing with heat waves, we have assigned the connected keywords of Fig. 5 to seven subject categories:

1. Countries/regions: United-States, Great-Plains
2. Cities: St-Louis, Athens, Chicago
3. Events: 1980 heat-wave, 1995 heat-wave
4. Impacts: impacts, responses, drought, precipitation, comfort, sultriness
5. Climate research: climate, climate change, model, temperature, variability
6. Medicine: cardiovascular deaths, mortality, air pollution
7. Meteorology: atmospheric flow, weather, heat, humidity index

### Important publications

**Fig. 6:**
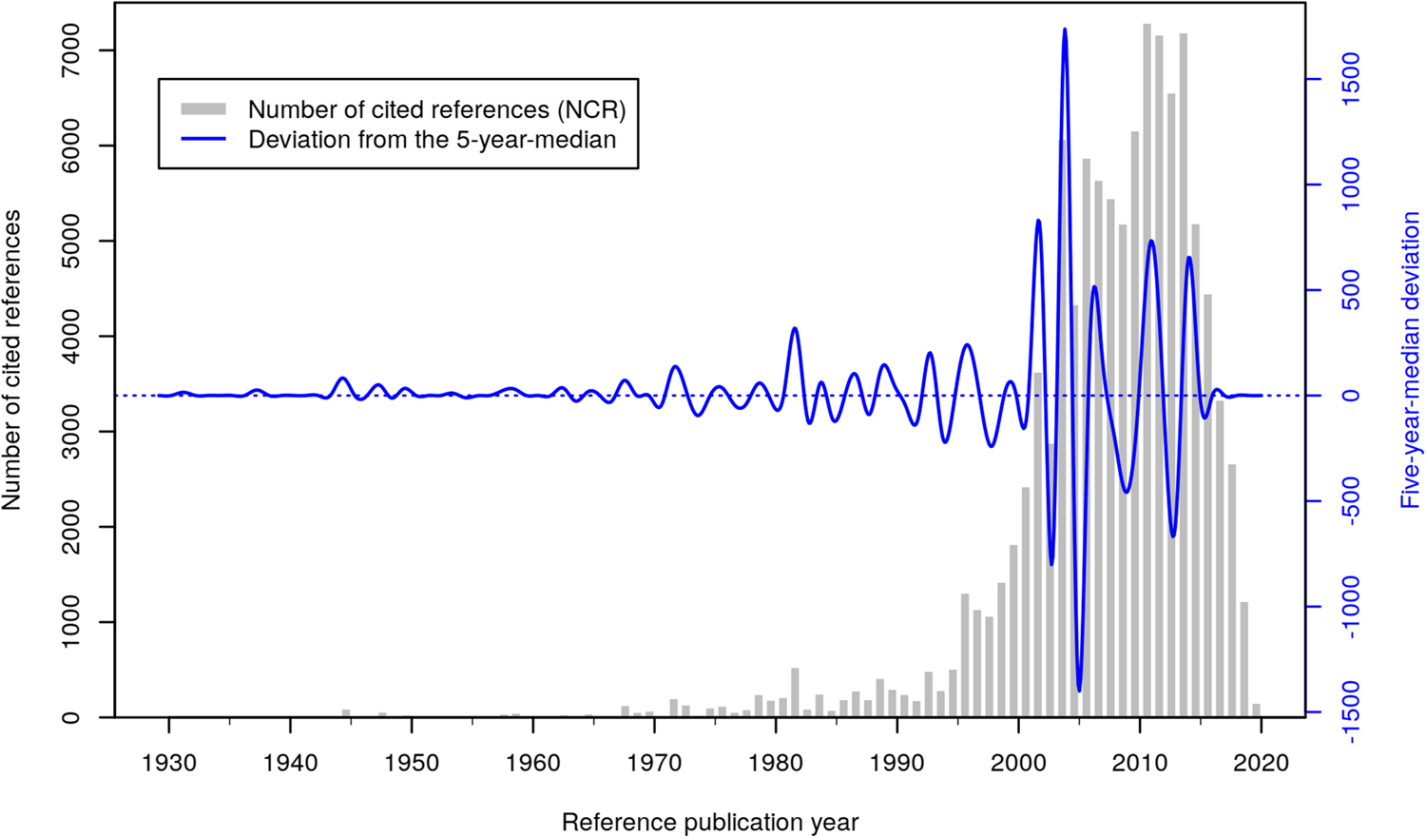
Annual distribution of cited references throughout the time period 1925–2020, which have been cited in heat wave-related papers (published between 1964 and 2020). Only references with a minimum reference count of 10 are considered

**Fig. 7:**
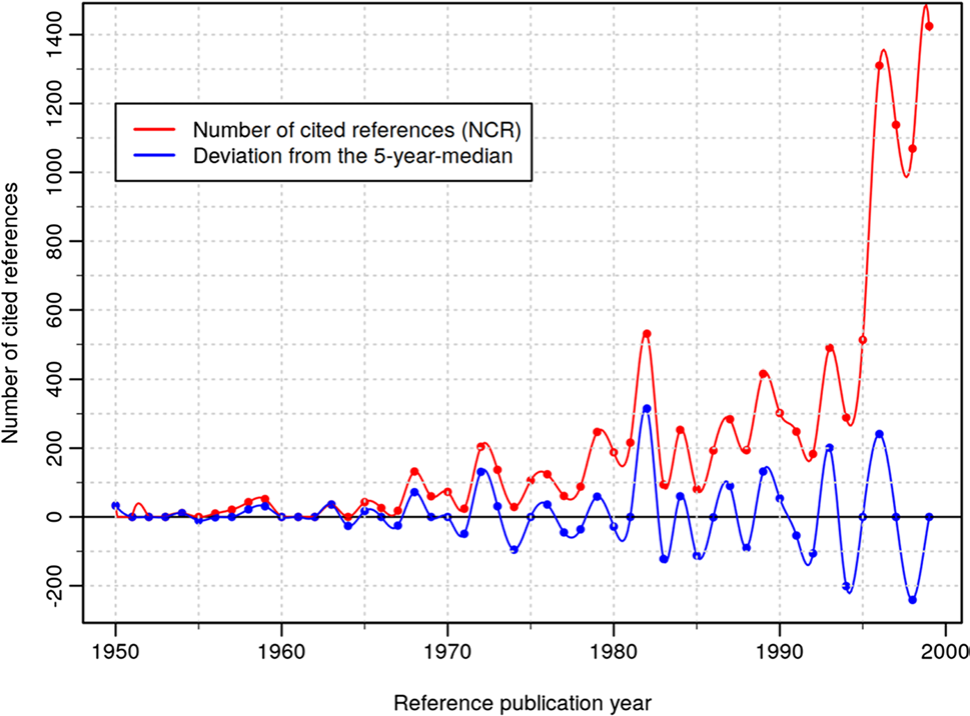
Annual distribution of cited references throughout the time period 1950–2000, which have been cited in heat wave-related papers (published between 1972 and 2020). Only references with a minimum reference count of 10 are considered

**Fig. 8:**
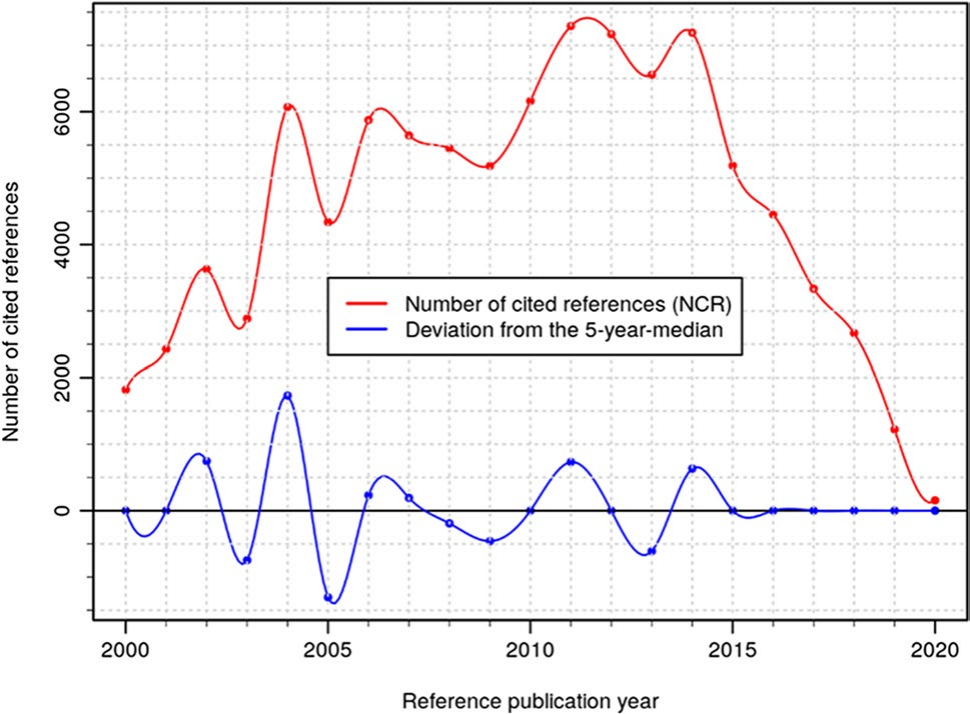
Annual distribution of cited references throughout the time period 2000–2020, which have been cited in heat wave-related papers (published between 2000 and 2020). Only references with a minimum reference count of 10 are considered

Figures 6–8 show the results of the RPYS analysis performed with the CRExplorer and present the distribution of the number of cited references across the reference publication years. Figure 6 shows the RPYS spectrogram of the full range of reference publication years since 1925. Figure 7 presents the spectrogram for the reference publication year period 1950–2000 for better resolving the historical roots. Figure 8 shows the spectrogram for the period 2000–2020, comprising the cited references from the bulk of the publication set analyzed.

The gray bars (Fig. 6) and red lines (Figs. 7–8) in the graphs visualize the number of cited references per reference publication year. In order to identify those publication years with significantly more cited references than other years, the (absolute) deviation of the number of cited references in each year from the median of the number of cited references in the two previous, the current, and the two following years (t − 2; t − 1; t; t + 1; t + 2) is also visualized (blue lines). This deviation from the 5-year median provides a curve smoother than the one in terms of absolute numbers. We inspected both curves for the identification of the peak papers.

Which papers are most important for the scientific community performing research on heat waves? We use the number of cited references (N_CR) as a measure of the citation impact within the topic-specific literature of our publication set. N_CR should not be confused with the overall number of citations of the papers as given by the WoS citation counts (times cited). These citation counts are based on all citing papers covered by the complete database (rather than a topic-specific publication set) and are usually much higher.

Applying the selection criteria mentioned above (minimum number of cited references between 50 and 150 in three different periods), 104 references have been selected as key papers (important papers most frequently referenced within the research topic analyzed) and are presented in Table 2 in Appendix 2. The peak papers corresponding to reference publication years below about 2000 can be seen as the historical roots of the research topic analyzed. Since around 2000, the number of references with the same publication year becomes increasingly numerous, usually with more than one highly referenced (cited) paper at the top. Although there are comparatively fewer distinct peaks visible in the RPYS spectrogram of Fig. 8, the most frequently referenced papers can easily be identified via the CRE reference listing. Depending on the specific skills and needs (i.e., the expert knowledge and the intended depth of the analysis), the number of top-referenced papers considered key papers can be defined individually.

Table 2 lists the first authors and titles of the 104 key papers selected, their number of cited references (N_CR), and the DOIs for easy access. Some N_CR values are marked by an asterisk, indicating a high value of the N_TOP10 indicator implemented in the CRExplorer. The N_TOP10 indicator value is the number of reference publication years in which a focal cited reference belongs to the 10% most referenced publications. In the case of about half of the cited references in Table 2 (*n* = 58), the N_TOP10 value exceeded a value of 9. The three highest values in our dataset are 24, 21, and 20.

**Fig. 9:**
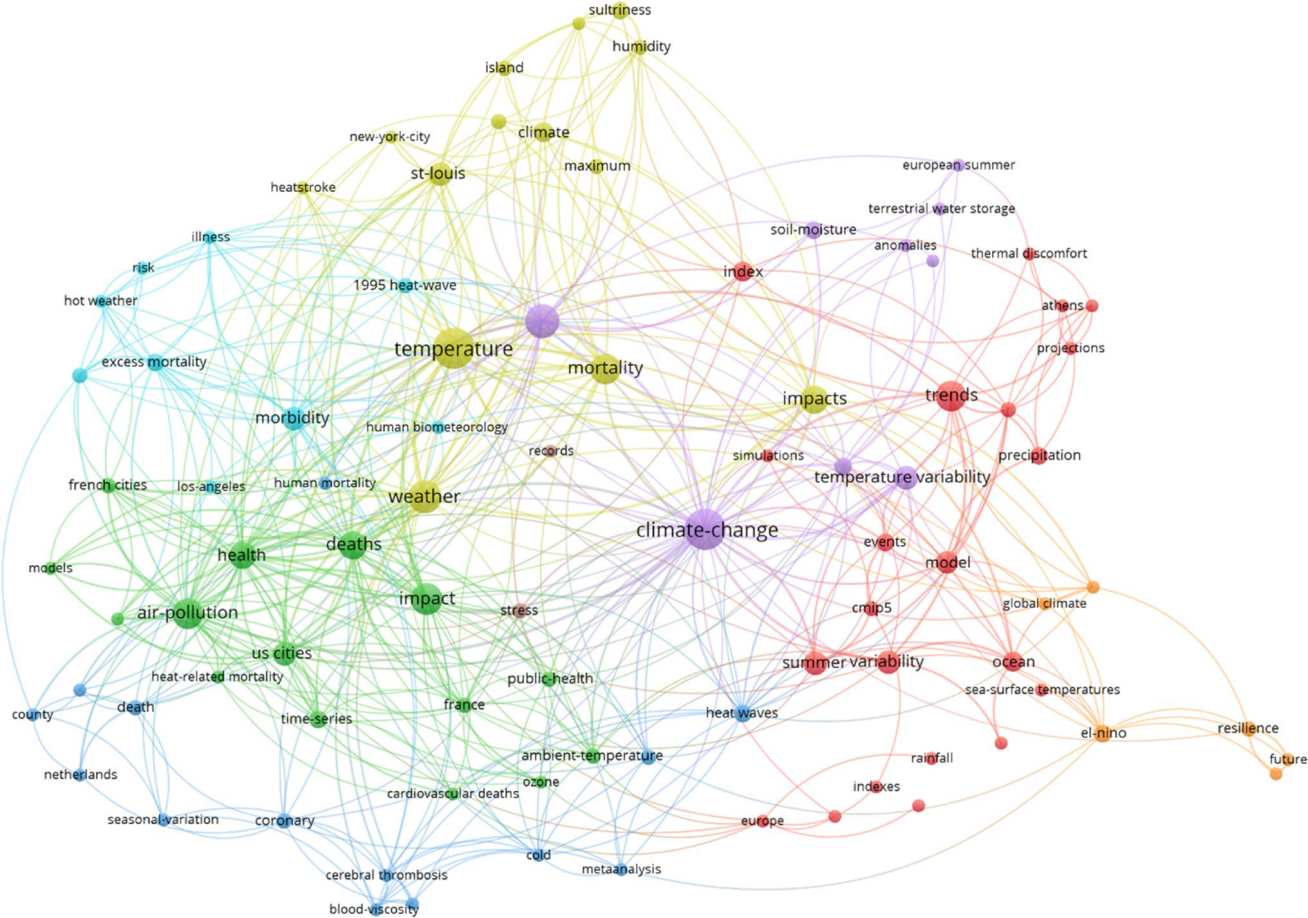
Co-occurrence network map of the keywords plus of the 104 key papers dealing with heat waves selected applying RPYS via CRE software and listed in Table 2. The minimum number of occurrences of keywords is 2; of the 310 keywords, 91 meet the threshold. Readers interested in an in-depth analysis can use VOSviewer interactively and zoom into the map via the following URL: https://tinyurl.com/4vwpc4t2

Out of the 104 key papers from Table 2, 101 have a DOI of which we found 101 papers in the WoS. Three papers have no DOI but could be retrieved from WoS. The altogether 104 papers were exported and their keywords (keywords plus) were displayed in Fig. 9 for revealing the thematic content of the key papers from the RPYS analysis at a glance.

Overall, the keywords mapped in Fig. 9 are rather similar to the keywords presented in Fig. 4. Besides climate change, temperature, weather, and air-pollution, the keywords deaths, health, mortality, and United-States appear as the most pronounced terms.

The key papers presented in Table 2 can be categorized as follows: (1) papers dealing with specific heat wave events, (2) the impact of heat waves on human health, (3) heat wave-related excess mortality and implications for prevention, (4) the interaction between air pollution and high temperature, (5) circulation pattern and the meteorological basis, (6) future perspectives and risks, and (7) climate models, indicators, and statistics.

## Discussion

Today, the hypothesis of a human-induced climate change is no longer abstract but has become a clear fact, at least for the vast majority of the scientific community (IPCC 2014; CSSR 2017; NCA4 2018; and the multitude of references cited therein). The consequences of a warmer climate are already obvious. The rapidly growing knowledge regarding the earth’s climate system has revealed the connection between global warming and extreme weather events. Heat waves impact people directly and tangibly and many people are pushing for political actions. Research on heat waves came up with the occurrence of some severe events in the second half of the twentieth century and was much stimulated by the more numerous, more intense, and longer lasting heat waves that have occurred since the beginning of the twenty-first century.

As already mentioned in Sect. 1, the more intense and more frequently occurring heat waves cannot be explained solely by natural climate variations but only with human-made climate change. As a consequence, research on heat waves has become embedded into meteorology and climate change research and has aimed to understand the specific connection with global warming. Scientists discuss a weakening of the polar jet stream as a possible reason for an increasing probability for the occurrence of heat waves (e.g., Broennimann et al. 2009; Coumou et al. 2015; Mann 2019). Climate models are used for projections of temperature and rainfall variability in the future, based on various scenarios of greenhouse gas emissions. As a result, the corresponding keywords appear in the maps of Figs. 4 and 9. Also, the application of statistics plays a major role in the papers of our publication set; some of the most highly referenced (early) papers in Table 2 primarily deal with statistical methods. These methods provide the basis for research on heat waves.

Our analysis shows that research on heat waves has become extremely important in the medical area, since severe heat waves have caused significant excess mortality (e.g., Kalkstein and Davis 1989; Fouillet et al. 2006; Anderson and Bell 2009, 2011). The most alarming is that the limit for survivability may be reached at the end of the twenty-first century in many regions of the world due to the fatal combination of rising temperatures and humidity levels (e.g., Pal and Eltahir 2016; Im et al. 2017; Kang and Eltahir 2018). The combination of heat and humidity is measured as the “wet-bulb temperature” (WBT), which is the lowest temperature that can be reached under current ambient conditions by the evaporation of water. At 100% relative humidity, the wet-bulb temperature is equal to the air temperature and is different at lower humidity levels. For example, an ambient temperature of 46 °C and a relative humidity of 50% correspond to 35 °C WBT, which is the upper limit that can kill even healthy people within hours. By now, the limit of survivability has almost been reached in some places. However, if global warming is not seriously tackled, deadly heat waves are anticipated for many regions that have contributed little to climate change.

According to high-resolution climate change simulations, North China and South Asia are particularly at risk, because the annual monsoon brings hot and humid air to these regions (Im et al. 2017; Kang and Eltahir 2018). The fertile plain of North China has experienced vast expansion of irrigated agriculture, which enhances the intensity of heat waves. South Asia, a region inhabited by about one-fifth of the global human population, is likely to approach the critical threshold by the late twenty-first century, if greenhouse gas emissions are not lowered significantly. In particular, the densely populated agricultural regions of the Ganges and Indus river basins are likely to be affected by extreme future heat waves. Also, the Arabic-speaking desert countries of the Gulf Region in the Middle East and the French-speaking parts of Africa are expected to suffer from heat waves beyond the limit of human survival. But to date, only 12 papers have been published on heat waves in connection with wet-bulb temperature (#15 of the search query); no paper was published before 2016. Some papers report excess hospital admissions during heat wave events (e.g., Semenza et al. 1999; Knowlton et al. 2009), with the danger of a temporary capacity overload of local medical systems in the future. Presumably, this will be an increasingly important issue in the future, when more and larger urban areas are affected by heat waves beyond the limit of human survival indicated by wet-bulb temperatures above 35° C.

The importance of heat waves for the medical area is underlined by the large portion of papers discussing excess hospital admissions and excess mortality during intense heat wave events, particularly in urban areas with a high population density. As was the case during the boom phase of the Covid-19 pandemic, local medical health care systems may become overstressed by long-lasting heat wave events and thus adaptation strategies are presented and discussed. Finally, the analysis of the keywords in this study reveals the connection of heat wave events with air pollution in urban regions. There seems to be evidence of an interaction between air pollution and high temperatures in the causation of excess mortality (e.g., Katsouyanni et al. 1993). Two more recent papers discuss the global risk of deadly heat (Mora et al. 2017) and the dramatically increasing chance of extremely hot summers since the 2003 European heat wave (Christidis et al. 2015).

Another important topic of the heat wave papers is related to the consequences for agriculture and forestry. Reduced precipitation and soil moisture result in crop failure and put food supplies at risk. Unfortunately, large regions of the world that contribute least to the emission of greenhouse gases are affected most by drought, poor harvests, and hunger. Some more recent papers discuss the increasing probability of marine heat waves (Oliver et al. 2018) and the consequences for the marine ecosystem (Smale et al. 2019).

The results of this study should be interpreted in terms of its limitations:

1. We tried to include in our bibliometric analyses all relevant heat wave papers covered by the database. Our long-standing experience in professional information retrieval has shown, however, that it is sheer impossible to get complete and clean results by search queries against the backdrop of the search functions provided by literature databases like WoS or others. Also, the transition from relevant to non-relevant literature is blurred and is a question of the specific needs. In this study, we used bibliometric methods that are relatively robust with regard to the completeness and precision of the publication sets analyzed. For example, it is an advantage of RPYS that a comparatively small portion of relevant publications (i.e., an incomplete publication set) contains a large amount of the relevant literature as cited references. The number of cited references is indeed lowered as a consequence of an incomplete publication set. However, this does not significantly affect the results, since the reference counts are only used as a relative measure within specific publication years.
2. As most literature databases, the WoS does not cover each and every scientific journal but only a carefully selected set of core journals most important for scientific disciplines. The coverage or comprehensiveness of the database can be estimated by comparing the number of all cited references with the number of the linked cited references (i.e., the references, which correspond to papers appearing in publications covered by the database as publication records). Based on the publication years 1990, 1995, 2000, 2005, and 2010, about 70% of all references in the natural sciences are linked references (Marx and Bornmann 2015). Thus, about 30% of the cited literature of these disciplines is not covered by the database in the form of paper records, presumably many non-English publications. It may be true that the publication set analyzed is biased toward mid-latitude developed countries, disadvantaging countries with most people suffering from humid heat waves. Parts of the most extreme heat waves occur in the French-speaking parts of Africa and the Arabic-speaking desert countries. Presumably, relevant literature like national reports discussing for example the local impact of extreme heat waves is not included in this analysis. However, if such documents were highly relevant, they should be cited in the literature covered by the WoS. In this case, our RPYS analysis would have discovered them. Therefore, we are confident that at least the highly relevant documents of the heat wave literature are considered in our analysis.
  Two other limitations of this study refer to the RPYS of the heat wave paper set:
3. There are numerous rather highly cited references retrieved by RPYS via CRExplorer but not considered in the listing of Table 2 due to the selection criteria applied. Many of these non-selected papers have N_CR values just below the limits that we have set. Therefore, papers not included in our listing are not per se qualified as much less important or even unimportant.
4. In the interpretation of cited references counts, one should have in mind that they rely on the “popularity” of a publication being cited in subsequent research. The counts measure impact but not scientific importance or accuracy (Tahamtan and Bornmann 2019). Note that there are many reasons why authors cite publications (Tahamtan and Bornmann 2018), thus introducing a lot of “noise” in the data (this is why RPYS focuses on the cited reference peaks).

Our suggestions for future empirical analysis refer to the impact of the scientific heat wave discourse on social networks and funding of basic research on heat waves around topics driven by political pressure. Whereas this paper focuses on the scientific discourse around heat waves, it would be interesting if future studies were to address the policy relevance of the heat waves research.

## Data Availability

Not applicable.

## Appendix 1 1)

WoS search query (date of search: July 1, 2021)

**Table:**

# 15	**12**	#14 AND #6*Indexes* = *SCI-EXPANDED, SSCI, A&HCI, CPCI-S, CPCI-SSH, BKCI-S, BKCI-SSH, ESCI Timespan* = *All years*
# 14	**1,293**	**TOPIC:** ("wet bulb temperature*" OR WBT) *Indexes* = *SCI-EXPANDED, SSCI, A&HCI, CPCI-S, CPCI-SSH, BKCI-S, BKCI-SSH, ESCI Timespan* = *All years*
# 13	**2,373**	#6 AND TS = mortality*Indexes* = *SCI-EXPANDED, SSCI, A&HCI, CPCI-S, CPCI-SSH, BKCI-S, BKCI-SSH, ESCI Timespan* = *All years*
# 12	**297**	#2 OR #4**Refined by:** **DOCUMENT TYPES:** ( ARTICLE OR MEETING ABSTRACT OR CORRECTION OR PROCEEDINGS PAPER OR LETTER OR REVIEW OR NEWS ITEM OR BOOK CHAPTER OR EARLY ACCESS OR EDITORIAL MATERIAL OR BOOK REVIEW) AND **PUBLICATION YEARS:** ( 1984 OR 1967 OR 1983 OR 1966 OR 1982 OR 1965 OR 1999 OR 1981 OR 1964 OR 1998 OR 1980 OR 1963 OR 1997 OR 1979 OR 1962 OR 1996 OR 1978 OR 1961 OR 1995 OR 1977 OR 1959 OR 1994 OR 1976 OR 1954 OR 1993 OR 1975 OR 1949 OR 1992 OR 1974 OR 1940 OR 1991 OR 1973 OR 1938 OR 1990 OR 1972 OR 1930 OR 1989 OR 1971 OR 1926 OR 1988 OR 1970 OR 1914 OR 1987 OR 1969 OR 1912 OR 1986 OR 1968 OR 1906 OR 1985)*Indexes* = *SCI-EXPANDED, SSCI, A&HCI, CPCI-S, CPCI-SSH, BKCI-S, BKCI-SSH, ESCI Timespan* = *All years*
# 11	**4,588**	#10 AND #6*Indexes* = *SCI-EXPANDED, SSCI, A&HCI, CPCI-S, CPCI-SSH, BKCI-S, BKCI-SSH, ESCI Timespan* = *All years*
# 10	**498,361**	#9 OR #8 OR #7*Indexes* = *SCI-EXPANDED, SSCI, A&HCI, CPCI-S, CPCI-SSH, BKCI-S, BKCI-SSH, ESCI Timespan* = *All years*
# 9	**191,712**	**TITLE:** (climat* OR palaeoclimat* OR paleoclimat*)*Indexes* = *SCI-EXPANDED, SSCI, A&HCI, CPCI-S, CPCI-SSH, BKCI-S, BKCI-SSH, ESCI Timespan* = *All years*
# 8	**121,301**	**TOPIC:** ("global temperature*" OR "global warm*" OR "greenhouse effect" OR "greenhouse gas*" OR "greenhouse warm*")*Indexes* = *SCI-EXPANDED, SSCI, A&HCI, CPCI-S, CPCI-SSH, BKCI-S, BKCI-SSH, ESCI Timespan* = *All years*
# 7	**331,878**	**TOPIC:** ("climate chang*" OR "climatic chang*" OR "climate varia*" OR "climatic varia*" OR "climate warm*" OR "climatic warm*")*Indexes* = *SCI-EXPANDED, SSCI, A&HCI, CPCI-S, CPCI-SSH, BKCI-S, BKCI-SSH, ESCI Timespan* = *All years*
# 6	**8,011**	#2 OR #4**Refined by:** **DOCUMENT TYPES:** ( ARTICLE OR MEETING ABSTRACT OR CORRECTION OR PROCEEDINGS PAPER OR LETTER OR REVIEW OR NEWS ITEM OR BOOK CHAPTER OR EARLY ACCESS OR EDITORIAL MATERIAL OR BOOK REVIEW)*Indexes* = *SCI-EXPANDED, SSCI, A&HCI, CPCI-S, CPCI-SSH, BKCI-S, BKCI-SSH, ESCI Timespan* = *All years*
# 5	**8,033**	#2 OR #4*Indexes* = *SCI-EXPANDED, SSCI, A&HCI, CPCI-S, CPCI-SSH, BKCI-S, BKCI-SSH, ESCI Timespan* = *All years*
# 4	**62**	#3 AND TS = (climat* OR greenhouse OR warming OR atmospher* OR tropospher* OR weather)*Indexes* = *SCI-EXPANDED, SSCI, A&HCI, CPCI-S, CPCI-SSH, BKCI-S, BKCI-SSH, ESCI Timespan* = *All years*
# 3	**597**	#1 NOT #2*Indexes* = *SCI-EXPANDED, SSCI, A&HCI, CPCI-S, CPCI-SSH, BKCI-S, BKCI-SSH, ESCI Timespan* = *All years*
# 2	**7,971**	**TOPIC:** ("heat wave" OR "heat waves" OR heatwave OR heatwaves OR "hot spell" OR "hot spells")**Refined by:** [excluding] **WEB OF SCIENCE CATEGORIES:** ( NANOSCIENCE NANOTECHNOLOGY OR ASTRONOMY ASTROPHYSICS OR NUCLEAR SCIENCE TECHNOLOGY OR PHYSICS APPLIED OR PHYSICS ATOMIC MOLECULAR CHEMICAL OR PHYSICS CONDENSED MATTER OR PHYSICS FLUIDS PLASMAS OR PHYSICS MATHEMATICAL OR PHYSICS MULTIDISCIPLINARY OR LITERARY REVIEWS OR MECHANICS)*Indexes* = *SCI-EXPANDED, SSCI, A&HCI, CPCI-S, CPCI-SSH, BKCI-S, BKCI-SSH, ESCI Timespan* = *All years*
# 1	**8,568**	**TOPIC:** ("heat wave" OR "heat waves" OR heatwave OR heatwaves OR "hot spell" OR "hot spells")*Indexes* = *SCI-EXPANDED, SSCI, A&HCI, CPCI-S, CPCI-SSH, BKCI-S, BKCI-SSH, ESCI Timespan* = *All years*

## Appendix 2

Table 2

**Table 2:** Listing of the key papers (*n* = 104) revealed by RPYS via CRE (*RPY* reference publication year, *N_CR* number of cited references, *Title* title of the cited reference, *DOI* allows easily to retrieve the full paper via WoS or Internet)

***RPY***	***N_CR***	***First author***	***Title***	***DOI***
1945	94	Mann, H.B	Nonparametric tests against trend	Not available
1968	108	Sen, P.K	Estimates of regression coefficient based on Kendalls Tau	Not available
1972	72	Schuman, S.H	Patterns of urban heat-wave deaths and implications for prevention—Data from New York and St-Louis during July, 1966	https://doi.org/10.1016/0013–9351(72)90,020–5
1973	81*	Oke, T.R	City size and urban heat island	https://doi.org/10.1016/0004–6981(73)90,140–6
1979	110*	Steadman, R.G	The assessment of sultriness. Part I: A temperature-humidity index based on human physiology and clothing science	https://doi.org/10.1175/1520–0450(1979)018<0861:TAOSPI>2.0.CO;2
1980	62	Berry, J	Photosynthetic response and adaptation to temperature in higher-plants	https://doi.org/10.1146/annurev.pp.31.060180.002423
1982	175	Oke, T.R	The energetic basis of the urban heat island	https://doi.org/10.1002/qj.49710845502
1982	107*	Jones, T.S	Morbidity and mortality associated with the July 1980 heat-wave in St.-Louis and Kansas-City, MO	https://doi.org/10.1001/jama.247.24.3327
1982	88	Kilbourne, E.M	Risk-factors for heat-stroke—A case–control study	https://doi.org/10.1001/jama.247.24.3332
1984	112*	Steadman, R.G	A universal scale of apparent temperature	https://doi.org/10.1175/1520–0450(1984)023<1674:AUSOAT>2.0.CO;2
1984	52	Mearns, L.O	Extreme high-temperature events—Changes in their probabilities with changes in mean temperature	https://doi.org/10.1175/1520–0450(1984)023<1601:EHTECI>2.0.CO;2
1986	59	Kalkstein, L.S	An evaluation of summer discomfort in the United-States using a relative climatological index	https://doi.org/10.1175/1520–0477(1986)067<0842:AEOSDI>2.0.CO;2
1986	53	Keatinge, W.R	Increased platelet and red-cell counts, blood-viscosity, and plasma-cholesterol levels during heat stress, and mortality from coronary and cerebral thrombosis	https://doi.org/10.1016/0002–9343(86)90,348–7
1987	71	Mayer, H	Thermal comfort of man in different urban environments	https://doi.org/10.1007/BF00866252
1989	87*	Kalkstein, L.S	Weather and human mortality—An evaluation of demographic and interregional responses in the United-States	https://doi.org/10.1111/j.1467–8306.1989.tb00249.x
1989	65	Dudhia, J	Numerical study of convection observed during the winter monsoon experiment using a mesoscale two-dimensional model	https://doi.org/10.1175/1520–0469(1989)046<3077:NSOCOD>2.0.CO;2
1989	53	Joseph, D.D	Heat waves	https://doi.org/10.1103/RevModPhys.61.41
1992	85*	Katz, R.W	Extreme events in a changing climate—Variability is more important than averages	https://doi.org/10.1007/BF00139728
1993	74*	Kunst, A.E	Outdoor air temperature and mortality in the Netherlands: A time-series analysis	https://doi.org/10.1093/oxfordjournals.aje.a116680
1993	52	Katsouyanni, K	Evidence for interaction between air-pollution and high-temperature in the causation of excess mortality	https://doi.org/10.1080/00039896.1993.9940365
1995	52	Sartor, F	Temperature, ambient ozone levels, and mortality during summer, 1994, in Belgium	https://doi.org/10.1006/enrs.1995.1054
1996	368*	Semenza, J.C	Heat-related deaths during the July 1995 heat wave in Chicago	https://doi.org/10.1056/NEJM199607113350203
1996	327*	Kalnay, E	The NCEP/NCAR 40-year reanalysis project	https://doi.org/10.1175/1520–0477(1996)077<0437:TNYRP>2.0.CO;2
1996	106*	Changnon, S.A	Impacts and responses to the 1995 heat wave: A call to action	https://doi.org/10.1175/1520–0477(1996)077<1497:IARTTH>2.0.CO;2
1996	84	Kunkel, K.E	The July 1995 heat wave in the midwest: A climatic perspective and critical weather factors	https://doi.org/10.1175/1520–0477(1996)077<1507:TJHWIT>2.0.CO;2
1996	63	Kalkstein, L.S	The Philadelphia hot weather-health watch warning system: Development and application, summer 1995	https://doi.org/10.1175/1520–0477(1996)077<1519:TPHWHW>2.0.CO;2
1997	180*	Whitman, S	Mortality in Chicago attributed to the July 1995 heat wave	https://doi.org/10.2105/AJPH.87.9.1515
1997	116*	Kalkstein, L.S	An evaluation of climate/mortality relationships in large US cities and the possible impacts of a climate change	https://doi.org/10.1289/ehp.9710584
1997	105*	Karl, T.R	The 1995 Chicago heat wave: How likely is a recurrence?	https://doi.org/10.1175/1520–0477(1997)078<1107:TCHWHL>2.0.CO;2
1997	74	Mlawer, E.J	Radiative transfer for inhomogeneous atmospheres: RRTM, a validated correlated-k model for the longwave	https://doi.org/10.1029/97JD00237
1997	72	Keatinge, W.R	Cold exposure and winter mortality from ischaemic heart disease, cerebrovascular disease, respiratory disease, and all causes in warm and cold regions of Europe	Not available
1997	53	Easterling, D.R	Maximum and minimum temperature trends for the globe	https://doi.org/10.1126/science.277.5324.364
1997	53	Mantua, N.J	A Pacific interdecadal climate oscillation with impacts on salmon production	https://doi.org/10.1175/1520–0477(1997)078<1069:APICOW>2.0.CO;2
1997	50	Ballester, F	Mortality as a function of temperature. A study in Valencia, Spain, 1991–1993	https://doi.org/10.1093/ije/26.3.551
1998	105*	Rooney, C	Excess mortality in England and Wales, and in Greater London, during the 1995 heatwave	https://doi.org/10.1136/jech.52.8.482
1998	81*	Dematte, J.E	Near-fatal heat stroke during the 1995 heat wave in Chicago	https://doi.org/10.7326/0003–4819-129–3-199,808,010–00,001
1998	55	Smoyer, K.E	A comparative analysis of heat waves and associated mortality in St. Louis, Missouri—1980 and 1995	https://doi.org/10.1007/s004840050082
1998	53	Gaffen, D.J	Increased summertime heat stress in the US	https://doi.org/10.1038/25030
1999	200*	Semenza, J.C	Excess hospital admissions during the July 1995 heat wave in Chicago	https://doi.org/10.1016/S0749-3797(99)00,025–2
1999	91	Hoppe, P	The physiological equivalent temperature—A universal index for the biometeorological assessment of the thermal environment	https://doi.org/10.1007/s004840050118
1999	56	Matzarakis, A	Applications of a universal thermal index: physiological equivalent temperature	https://doi.org/10.1007/s004840050119
1999	52*	Kunkel, K.E	Temporal fluctuations in weather and climate extremes that cause economic and human health impacts: A review	https://doi.org/10.1175/1520–0477(1999)080<1077:TFIWAC>2.0.CO;2
2000	275*	Easterling, D.R	Climate extremes: observations, modelling, and impacts	https://doi.org/10.1126/science.289.5487.2068
2001	277*	Robinson, P.J	On the definition of a heat wave	https://doi.org/10.1175/1520–0450(2001)040<0762:OTDOAH>2.0.CO;2
2001	203*	Hynen, M.M.T.E	The impact of heat waves and cold spells on mortality rates in the Dutch population	https://doi.org/10.2307/3454704
2002	286*	Basu, R	Relation between elevated ambient temperature and mortality: A review of the epidemiologic evidence	https://doi.org/10.1093/epirev/mxf007
2002	279*	Curriero, F.C	Temperature and mortality in 11 cities of the eastern United States	https://doi.org/10.1093/aje/155.1.80
2002	233*	Frich, P	Observed coherent changes in climatic extremes during the second half of the twentieth century	https://doi.org/10.3354/cr019193
2002	156*	Bouchama, A	Medical progress—Heat stroke	https://doi.org/10.1056/NEJMra011089
2004	1099*	Meehl, G.A	More intense, more frequent, and longer lasting heat waves in the twenty-first century	https://doi.org/10.1126/science.1098704
2004	562*	Schaer, C	The role of increasing temperature variability in European summer heatwaves	https://doi.org/10.1038/nature02300
2004	326*	Stott, P.A	Human contribution to the European heatwave of 2003	https://doi.org/10.1038/nature03089
2004	232*	Beniston, P.M	The 2003 heat wave in Europe: A shape of things to come? An analysis based on Swiss climatological data and model simulations	https://doi.org/10.1029/2003GL018857
2004	209*	Black, E	Factors contributing to the summer 2003 European heatwave	https://doi.org/10.1256/wea.74.04
2004	180*	Luterbacher, J	European seasonal and annual temperature variability, trends, and extremes since 1500	https://doi.org/10.1126/science.1093877
2004	152	Kovats, R.S	Contrasting patterns of mortality and hospital admissions during hot weather and heat waves in Greater London, UK	https://doi.org/10.1136/oem.2003.012047
2005	313*	Ciais, P	Europe-wide reduction in primary productivity caused by the heat and drought in 2003	https://doi.org/10.1038/nature03972
2005	161*	Patz, J.A	Impact of regional climate change on human health	https://doi.org/10.1038/nature04188
2005	157*	Conti, S	Epidemiologic study of mortality during the Summer 2003 heat wave in Italy	https://doi.org/10.1016/j.envres.2004.10.009
2006	298*	Fouillet, A	Excess mortality related to the August 2003 heat wave in France	https://doi.org/10.1007/s00420-006–0089-4
2006	257*	Alexander, L.V	Global observed changes in daily climate extremes of temperature and precipitation	https://doi.org/10.1029/2005JD006290
2006	190*	Seneviratne, S.I	Land–atmosphere coupling and climate change in Europe	https://doi.org/10.1038/nature05095
2006	180*	Vandentorren, S	August 2003 heat wave in France: Risk factors for death of elderly people living at home	https://doi.org/10.1093/eurpub/ckl063
2006	177*	Hajat, S	Impact of high temperatures on mortality—Is there an added heat wave effect?	https://doi.org/10.1097/01.ede.0000239688.70829.63
2006	153*	Kovats, R.S	Heatwaves and public health in Europe	https://doi.org/10.1093/eurpub/ckl049
2007	238*	Fischer, E.M	Soil moisture—Atmosphere interactions during the 2003 European summer heat wave	https://doi.org/10.1175/JCLI4288.1
2007	156*	Della-Marta, P.M	Doubled length of western European summer heat waves since 1880	https://doi.org/10.1029/2007JD008510
2008	372*	Robine, J.M	Death toll exceeded 70,000 in Europe during the summer of 2003	https://doi.org/10.1016/j.crvi.2007.12.001
2008	287*	Kovats, R.S	Heat stress and public health: A critical review	https://doi.org/10.1146/annurev.publhealth.29.020907.090843
2008	178*	Baccini, M	Heat effects on mortality in 15 European cities	https://doi.org/10.1097/EDE.0b013e318176bfcd
2008	175*	Luber, G	Climate change and extreme heat events	https://doi.org/10.1016/j.amepre.2008.08.021
2008	160*	Fouillet, A	Has the impact of heat waves on mortality changed in France since the European heat wave of summer 2003? A study of the 2006 heat wave	https://doi.org/10.1093/ije/dym253
2009	339*	Anderson, Brooke G	Weather-related mortality: How heat, cold, and heat waves affect mortality in the United States	https://doi.org/10.1097/EDE.0b013e318190ee08
2009	212*	Basu, R	High ambient temperature and mortality: A review of epidemiologic studies from 2001 to 2008	https://doi.org/10.1186/1476-069X-8–40
2009	205*	Knowlton, K	The 2006 California heat wave: Impacts on hospitalizations and emergency department visits	https://doi.org/10.1289/ehp.11594
2010	282*	Fischer, E.M	Consistent geographical patterns of changes in high-impact European heatwaves	https://doi.org/10.1038/NGEO866
2010	208*	D’Ippoliti, D	The impact of heat waves on mortality in 9 European cities: results from the EuroHEAT project	https://doi.org/10.1186/1476-069X-9–37
2010	208*	Seneviratne, S.I	Investigating soil moisture-climate interactions in a changing climate: A review	https://doi.org/10.1016/j.earscirev.2010.02.004
2010	196*	Garcia-Herrera, R	A review of the European summer heat wave of 2003	https://doi.org/10.1080/10643380802238137
2011	381*	Dee, D.P	The ERA-Interim reanalysis: configuration and performance of the data assimilation system	https://doi.org/10.1002/qj.828
2011	355*	Barriopedro, D	The hot summer of 2010: Redrawing the temperature record map of Europe	https://doi.org/10.1126/science.1201224
2011	303*	Anderson, G.B	Heat waves in the United States: Mortality risk during heat waves and effect modification by heat wave characteristics in 43 U.S. communities	https://doi.org/10.1289/ehp.1002313
2011	198*	Dole, R	Was there a basis for anticipating the 2010 Russian heat wave?	https://doi.org/10.1029/2010GL046582
2011	154*	Gasparrini, A	The Impact of Heat Waves on Mortality	https://doi.org/10.1097/EDE.0b013e3181fdcd99
2012	243	Taylor, K.E	An overview of CMIP5 and the experiment design	https://doi.org/10.1175/BAMS-D-11–00,094.1
2012	236	Perkins, S.E	Increasing frequency, intensity and duration of observed global heatwaves and warm spells	https://doi.org/10.1029/2012GL053361
2012	204	Coumou, D	A decade of weather extremes	https://doi.org/10.1038/NCLIMATE1452
2013	289	Perkins, S.E	On the measurement of heat waves	https://doi.org/10.1175/JCLI-D-12–00,383.1
2013	159	Wernberg, T	An extreme climatic event alters marine ecosystem structure in a global biodiversity hotspot	https://doi.org/10.1038/NCLIMATE1627
2014	197	Miralles, D.G	Mega-heatwave temperatures due to combined soil desiccation and atmospheric heat accumulation	https://doi.org/10.1038/NGEO2141
2014	171	Russo, S	Magnitude of extreme heat waves in present climate and their projection in a warming world	https://doi.org/10.1002/2014JD022098
2015	182	Gasparrini, A	Mortality risk attributable to high and low ambient temperature: A multicountry observational study	https://doi.org/10.1016/S0140-6736(14)62,114–0
2015	168	Perkins, S.E	A review on the scientific understanding of heatwaves—Their measurement, driving mechanisms, and changes at the global scale	https://doi.org/10.1016/j.atmosres.2015.05.014
2015	159	Russo, S	Top ten European heatwaves since 1950 and their occurrence in the coming decades	https://doi.org/10.1088/1748–9326/10/12/124003
2015	131	Bond, N.A	Causes and impacts of the 2014 warm anomaly in the NE Pacific	https://doi.org/10.1002/2015GL063306
2015	111	Christidis, N	Dramatically increasing chance of extremely hot summers since the 2003 European heatwave	https://doi.org/10.1038/NCLIMATE2468
2016	197	Hobday, A.J	A hierarchical approach to defining marine heatwaves	https://doi.org/10.1016/j.pocean.2015.12.014
2016	121	Wernberg, T	Climate-driven regime shift of a temperate marine ecosystem	https://doi.org/10.1126/science.aad8745
2016	113	Di Lorenzo, E.	Multi-year persistence of the 2014/15 North Pacific marine heatwave	https://doi.org/10.1038/NCLIMATE3082
2017	125	Mora, C	Global risk of deadly heat	https://doi.org/10.1038/NCLIMATE3322
2017	112	Hughes, T.P	Global warming and recurrent mass bleaching of corals	https://doi.org/10.1038/nature21707
2018	209	Oliver, E.C.J	Longer and more frequent marine heatwaves over the past century	https://doi.org/10.1038/s41467-018–03,732-9
2018	124	Frolicher, T.L	Marine heatwaves under global warming	https://doi.org/10.1038/s41586-018–0383-9
2019	126	Smale, D.A	Marine heatwaves threaten global biodiversity and the provision of ecosystem services	https://doi.org/10.1038/s41558-019–0412-1

*N_TOP10 > 9; the N_TOP10 indicator is the number of reference publication years in which a focal cited reference belongs to the 10% most referenced publications.
